# Supplementary material for: Content-rich biological network constructed by mining PubMed abstracts
Source: BMC Bioinformatics. 2004 Oct 8;5:147. doi: 10.1186/1471-2105-5-147 (PMC528731; doi:10.1186/1471-2105-5-147)
Supplement: Additional File 2 — The original results of the above study (non-essential files are deleted to keep the file size under the limit set by BMC bioinformatics). [file 1471-2105-5-147-S2.bz2 › chilibotAdditionalFile2/dip30/644ID8642594E11031/html/F_G.html]

 


 **F** and **G** 
  
Found 14344 abstracts in PubMed, retrieved 30.  
 

 What does Google say? 
 PDF only 
| .edu only 

---

**Interactive relationship** (e.g. stimulation, inhibition, etc)

**Neutral relationship**- Mice died from GVHD in group  **G**  were significantly more than those in group E and group  **F**  p <.  Ref: 12864951 Zhonghua Xue Ye Xue Za Zhi, 2003
- compared with 14 C FIAU, 18  **F**  FHBG led to as high %ID  **g**  in HSV1 tk expressing hepatocytes and to significantly greater %ID  **g**  in C6 stb sr39tk xenografts and HSV1 sr39tk expressing hepatocytes.  Ref: 12856156 Eur J Nucl Med Mol Imaging< MedlineTA>Eur J Nucl Med Mol Imaging, 2003
- SpoIIAB, an anti sigma factor involved in regulation of sigma  **F** , is also shown to form a complex with sigma  **G**  in vitro.  Ref: 12867473 J Bacteriol, 2003
- The  **F**  tra region encodes eight of the 10 highly conserved core gene products of T4SS including TraA  **F**  pilin, the TraB  **F** , K  **F**  secretin like, V  **F**  lipoprotein and TraC  **F**  NTPase, E  **F** , L  **F**  and TraG  **F**  N terminal region which correspond to TrbC P, I P,  **G**  P, H P, E P, J P, D P and TrbL P, respectively, of the P type T4SS exemplified by the IncP plasmid RP4.  Ref: 12855161 FEMS Microbiol Lett, 2003

**Non-interactive relationship** (e.g. studied together, co-existance, homology, etc.)

- Zoccali C, Mallamaci  **F** , Tripepi  **G** , Cutrupi S, Parlongo S, Malatino LS, Bonanno  **G** , Rapisarda  **F** , Fatuzzo P, Seminara  **G** , Stancanelli B, Nicocia  **G** , Buemi M Institute of Biomedicine, Clinical Epidemiology and Pathophysiology of Renal Diseases and Hypertension, Reggio Cal.  Ref: 12859694 J Intern MedJ Intern Med, 2003
- We report here the generation of a soluble form of TXAS by deletion of the amino terminal membrane anchoring domain and replacement of the helix  **F**  and  **F**   **G**  loop region with the corresponding region of the structurally characterized microsomal P450 2C5.  Ref: 12859980 Arch Biochem Biophys, 2003
- Molecular cloning identified six causative genes FANCA, C, D2, E,  **F** , and  **G**  encoding a multiprotein complex whose precise biochemical function remains elusive.  Ref: 12861027 Mol Cell BiolMol Cell Biol, 2003
- Compared with traditional heating, central heating significantly increases the risk of exposure to Der  **f**  1 levels > ; 2 μ **g**   **g**  of dust odds ratio, 7.35.  Ref: 12859558 AllergyAllergy, 2003
- The ability of bPIV3 to function as a virus vaccine vector was explored further by introducing the RSV attachment  **G**  and fusion  **F**  genes into the bPIV3 RNA genome.  Ref: 12867647 J Gen Virol, 2003
- 14 C FIAU led to significantly higher %ID  **g**  in C6 stb tk xenografts than 18  **F**  FHBG P< ; 0.05.  Ref: 12856156 Eur J Nucl Med Mol Imaging< MedlineTA>Eur J Nucl Med Mol Imaging, 2003
- Over 2, and 16 wk, on alternate days, mice were treated with an intraperitoneal injection of either 1  **g** .kg 1.day 1 of human intact immunoglobulin or  **F**  ab 2 fragments of human immunoglobulin.  Ref: 12860569 Am J Physiol Heart Circ Physiol, 2003
- This recombinant virus expressed the RSV  **G**  and  **F**  proteins sufficiently to evoke a protective immune response in hamsters upon challenge with RSV or human PIV3 and to elicit RSV neutralizing and PIV3 haemagglutinin inhibition serum antibodies.  Ref: 12867647 J Gen Virol, 2003
- Given the recognized role of proinflammatory cytokines in septic shock and of extracellular  **F**  and  **G**  actin in the pathophysiology of multiple organ dysfunction, we have investigated the role of Tbeta 4 in sepsis.  Ref: 12860178 Int ImmunopharmacolInt Immunopharmacol, 2003
- Some optimal physicochemical characteristics e. **g** . solvent exposed area, solubility of the molecule may be responsible for this special feature of  **f**  MLP at such a low level of phylogeny.  Ref: 12867161 Cell Biol Int, 2003
- Since three of these genera Finegoldia, Micromonas and Peptostreptococcus contain only a single species,  **F** . magna, M. micros and P. anaerobius, respectively, these organisms were identified to the species level directly by using the multiplex PCR  **G** .  Ref: 12855723 Microbiology< MedlineTA>Microbiology, 2003
- Group A received no treament, group B received TBI only, the rest groups were irradiated 9 Gy by 60 Co and transfused 10 5 CD 34 cells or mixed with 10 7 E, 10 8  **F** , 1.5 x 10 8  **G**  of CD 3 cells respectively.  Ref: 12864951 Zhonghua Xue Ye Xue Za Zhi, 2003
